# Evaluation of a facility-based inspection tool to assess lymphedema management services in Vietnam

**DOI:** 10.1371/journal.pntd.0008773

**Published:** 2020-10-19

**Authors:** Do Trung Dung, Vu Thi Lam Binh, Caitlin M. Worrell, Molly Brady, Victoria Walsh, Aya Yajima, Zeina Sifri, LeAnne M. Fox

**Affiliations:** 1 National Institute of Malariology, Parasitology, and Entomology, Hanoi, Vietnam; 2 U.S. Centers for Disease Control and Prevention, Atlanta, Georgia, United States of America; 3 Swiss Tropical and Public Health Institute (Swiss TPH), Basel, Switzerland; 4 University of Basel, Basel, Switzerland; 5 RTI International, Washington, D.C., United States of America; 6 World Health Organization Western Pacific Regional Office, Manila, Philippines; 7 Helen Keller International, Washington, D.C., United States of America; NIH-NIRT-ICER, INDIA

## Abstract

Assuring availability of services for patients with lymphedema is required for countries to be validated as having achieved elimination of lymphatic filariasis (LF). A direct inspection protocol (DIP) tool, designed to measure the readiness to provide quality lymphedema management services, has recently been developed. The DIP tool includes 14 indicators across six quality themes: trained staff, case management and education materials, water infrastructure, medicines and commodities, patient tracking system, and staff knowledge. We evaluated the use of the tool in Vietnam, where data were needed to inform validation efforts. To apply the tool in Vietnam, we compiled a list of 219 commune health stations (CHS) with known lymphedema patients and conducted a cross-sectional survey in 32 CHS; including 24 in Red River Delta region, 2 in the North Central region, and 6 in the South Central Coast region. The mean facility score, calculated by assigning 1 point per indicator, was 8.8 of 14 points (63%, range 4[29%]-13[93%]). Percentage of surveyed facilities with staff trained in last two years was 0%; availability of lymphedema management guidelines (56%); availability of information, education, and communication materials (16%); reliable improved water infrastructure (94%); availability of antiseptics (81%), antifungals (44%), analgesics or anti-inflammatories (97%), antibiotics (94%); supplies for lymphedema and acute attack management (100%); lymphedema patients recorded in last 12 months (9%); staff knowledge about lymphedema signs/symptoms (63%), lymphedema management strategies (72%), signs/symptoms of acute attacks (81%), and acute attack management strategies (75%). The tool allowed standardized assessment of readiness to provide quality services. Lack of trained health staff, limited patient tracking, and depletion of education materials were identified as challenges and addressed by the national program. Survey data were included in the validation dossier, providing evidence necessary for WHO to validate Vietnam as having eliminated lymphatic filariasis in 2018.

## Author summary

Lymphedema, a chronic swelling of the limbs, is a consequence of a debilitating and disfiguring neglected parasitic infection known as lymphatic filariasis (LF). In places where LF is common, the World Health Organization (WHO) recommends a simple and effective hygiene program to improve the quality of life of people living with lymphedema. In places where persons with lymphedema are found, health facilities should have the ability to teach these simple techniques and manage complications. Further, WHO recommends that countries evaluate their lymphedema care services to ensure quality and requires information about lymphedema care when deciding whether to acknowledge that a country has eliminated LF. We developed a tool for use in assessing readiness of health facilities to provide quality lymphedema care, then evaluated the tool in health facilities across three regions of Vietnam, collecting information on 14 factors related to lymphedema care quality. Most facilities had needed supplies and the staff generally understood how to care for persons with lymphedema; however, facilities often lacked patient tracking systems or health education materials. Vietnam used this information to improve lymphedema care services throughout the country and received acknowledgement of LF elimination from WHO in 2018.

## Introduction

Lymphatic filariasis (LF) is a parasitic infection caused by filarial nematodes transmitted by mosquitoes. Lymphatic filariasis is characterized by both chronic manifestations and acute exacerbations of disease. The two most common chronic clinical manifestations are lymphedema—fluid retention due to a compromised lymphatic system that usually affects the legs, but may also occur in the arms, breast, or genital region—and hydrocele, or scrotal swelling [1]. Acute attacks, or episodes of adenolymphangitis, occur when patients with damaged lymphatic systems develop skin lesions that lead to secondary bacterial infections [2]. These acute attacks are responsible for further progression of lymphedema and can result in impairment, activity limitations, restrictions to participation, and critical losses to productivity [3].

In 1997, the World Health Assembly endorsed Resolution 50.29, calling upon Member States to develop national plans to eliminate LF [4]. Subsequently, the World Health Organization (WHO) established the Global Programme to Eliminate Lymphatic Filariasis (GPELF) in 2000. The GPELF is founded on two pillars: i) interruption of transmission through mass drug administration (MDA) and ii) alleviation of suffering through ensuring morbidity management and disability prevention (MMDP) services are available in all areas with persons with lymphedema or hydrocele [5].

To receive acknowledgement as achieving elimination of LF as a public health problem, national programs must submit dossiers to WHO with evidence supporting achievement of elimination targets, including an assessment of the quality of lymphedema management services in health facilities designated to provide care to lymphedema patients [6]. WHO recommended a quality assessment as part of dossier criteria to provide information to national programs on the strengths and weaknesses of the lymphedema services and to identify areas where improvements may be needed to ensure quality services are available to all patients. The dossier also recommends assessment of hydrocelectomy services, where applicable; however, this study focuses only on the assessment of lymphedema and adenolymphangitis management services.

The WHO and partners recently developed the direct inspection protocol (DIP), a tool to assess the readiness of health facilities to provide quality lymphedema and adenolymphangitis services [7]. The DIP includes a set of indicators, modeled on the WHO Service Availability and Readiness Assessment tool (SARA) and includes indicators from WHO/UNICEF guide to monitor water, sanitation and hygiene in health care facilities as well as indicators specific to lymphedema and adenolymphangitis management [8, 9], chosen through a Delphi expert consultation in December 2015 [10]. The protocol includes a standardized questionnaire designed to capture 14 equally weighted tracer indicators grouped into six quality domains (Table 1). In addition to the standardized questionnaire, there are two sets of semi-structured questions that can be used to collect qualitative information from health staff on challenges with lymphedema management service delivery and from patients on their knowledge of lymphedema management, health status, and quality of life.

**Table 1:** Quality domains and tracer indicators included in the direct inspection protocol questionnaire.

Quality domains	Tracer indicators
Trained staff	1. At least one facility staff member trained in lymphedema management in the last two years;
Case management and education materials	2. At least one guideline for lymphedema management is present at the health facility; 3. At least one information, education, and communication (IEC) awareness material for lymphedema management is present at the facility;
Water infrastructure	4. The main water for the facility is an improved source, is located on the premises, and is functional at the time of the visit [9];
Medicines and commodities	5. Antiseptics (e.g. potassium permanganate or other anti-bacterial) are present at the facility; 6. Antifungals (e.g. potassium permanganate or Whitfield’s ointment) are present at the facility; 7. Oral/injectable antibiotics are available at the facility; 8. Analgesics (e.g. paracetamol) are present at the facility; 9. At least one supply for lymphedema and acute attack management is available at the facility;
Patient tracking system	10. A system for patient tracking with at least one patient recorded in the last 12 months;
Staff knowledge	11. Clinic staff member able to correctly identify at least two signs or symptoms of lymphedema; 12. Clinic staff member able to correctly identify at least two lymphedema management strategies; 13. Clinic staff member able to correctly identify at least two signs or symptoms of an acute attack; 14. Clinic staff member able to correctly identify at least two strategies to treat a patient with an acute attack.

The protocol recommends randomly selecting at least 10% of health facilities providing services for lymphedema from a list of all facilities designated to provide lymphedema management services nationally [6]. However, countries can select additional facilities to meet local needs; for example, certain health facilities can also be purposefully selected in areas of highest burden or where concerns exist about implementation of services.

Following aggregation of information across the 14 quality indicators, summary scores are calculated for each facility and each indicator. While the protocol does not specify a passing score, scores can be used to identify health facilities and indicators that could be targeted to improve quality of health services. Suggested follow-up actions are provided by WHO for each domain (S1 Table). Recognizing that many of the domains fall outside the mandate of the LF national program, many of the actions involve sharing the data with other units within the Ministry of Health and other ministries to advocate for service delivery improvements within the context of health systems strengthening.

### LF in Vietnam

Within the Ministry of Health (MOH), the National Institute for Malariology, Parasitology and Entomology (NIMPE) is responsible for direction and implementation of the LF elimination program, while the commune health stations (CHS) are responsible for lymphedema management and MDA.

In Vietnam, LF in the North Central and South Central Coast regions was caused by the parasitic roundworm species *Wuchereria bancrofti*, whereas in the North Central and Red River Delta regions, it was caused by *Brugia malayi* [11]. Surveys in the 1960s-1990s identified transmission in 24 provinces; however, LF transmission began declining in the 1960s, likely due to socio-ecological changes and treatment of microfilaremic individuals with diethylcarbamazine citrate (DEC) [11]. By 2002, only six districts (implementation units or IUs) required MDA, namely Binh Luc district (Ha Nam province) and Phu Cu (Hung Yen province) in the Red River Delta (RRD) region; and Khanh Vinh, Dien Khanh, Ninh Hoa (Khanh Hoa province) and Bac Ai (Ninh Thuan province) in the South Central Coast [12].

Since 2002, there have been various efforts by the national LF program to collect information about cases of lymphedema from CHS in districts and provinces with known historical transmission of LF. Based on these data, the national program led an LF MMDP management training in 2005 for provincial and CHS staff in the four provinces with highest numbers of known patients, all in the RRD region; patients were trained and provided self-care kits to promote the uptake of lymphedema self-care. As of the end of 2015, post-MDA surveillance surveys had finished in all districts with prior MDA, and Vietnam had been advised to submit a dossier for validation of elimination.

The objectives of this activity were i) to evaluate the use of the DIP tool and ii) to collect data needed to document implementation of MMDP services required for the validation of elimination of LF as a public health problem in Vietnam.

## Methods

### Ethics statement

This activity was considered program evaluation by NIMPE. Further, this activity was approved as non-research program evaluation by the Office of the Associate Director for Science, Center for Global Health, Centers for Disease Control and Prevention (protocol #2016–047). Prior to survey implementation, all patients and CHS were notified about the survey by province and district staff. At the time of survey, all patients and health staff were asked to provide verbal consent prior to the interview. All recruited patients were adults. Interviews with health staff were conducted privately, without supervisors present.

### Selection of health facilities

CHS staff provided sex, age, and location of known patients with lymphedema to NIMPE. This information was used to develop a sampling frame that included all CHS with known lymphedema patients in all areas with a history of LF. Due to the difference in data availability and the methods used to estimate the number of lymphedema patients, the sampling in Vietnam was operationalized differently in the three geographic regions. In the RRD region, patient estimates were available by CHS from a 2012 survey in which some CHS staff went door-to-door to search for patients, while other facilities reported patients identified from clinic records. Patients reported during this survey were not all validated as having lymphedema by medical staff. Per WHO recommendations, 22 facilities, representing approximately 10% of the 213 facilities with known lymphedema patients in the RRD region, were randomly selected. Two additional CHS in the RRD were purposely selected as they had higher numbers of known patients, (24 and 27 patients in Chau Giang CHS and Yen Bac CHS respectively), compared to the usual one or two patients in other CHS. In Quang Binh province, which provided a list of known patients to NIMPE in early 2016, the two CHS with known patients were selected. Within the Khanh Hoa and Ninh Thuan provinces in the South Central Coast region, patients were identified from four CHS during MDA data collection activities in 2005. While NIMPE attempted to update these data, not all CHS in the South Central Coast region responded to a request for information on the number of lymphedema patients by the time of the DIP implementation. Therefore, two additional CHS from the two districts that had conducted MDA in these provinces were selected randomly; for a total of 6 CHS.

### Data collection

Data collection took place in March and April 2016. At each selected health facility, a data collection team used a standardized questionnaire to collect information on the 14 quality indicators through key informant interviews with health staff. Further, on the day of the visit, data collection teams physically verified the presence of medicines and supplies necessary to treat lymphedema and adenolymphangitis. Semi-structured questions were included to solicit feedback from commune CHS staff related to improving MMDP service quality. Where possible, a patient at each CHS was also interviewed.

Data were collected by a team of one main surveyor from NIMPE, as well as other members from provincial health departments. Interview and visual inspection data were entered directly into Android phones, preloaded with electronic questionnaires, and uploaded by using the CommcareHQ platform (Dimagi Inc., Cambridge, MA).

### Statistical analysis

Scores were generated for each CHS surveyed, and a national score was generated for each tracer indicator using SAS (v9.4, Cary, NC). At each facility, if all criteria were met the indicator was marked as 1, otherwise the indicator was marked as 0. Scores for facilities were calculated based on equally-weighting all 14 indicators, ranging from 0 (lowest performing) to 14 (highest performing). National scores were calculated as the proportion of facilities meeting the indicator criteria. Results from semi-structured interviews with commune health staff and patients were analyzed for common themes.

## Results

**Fig 1:**
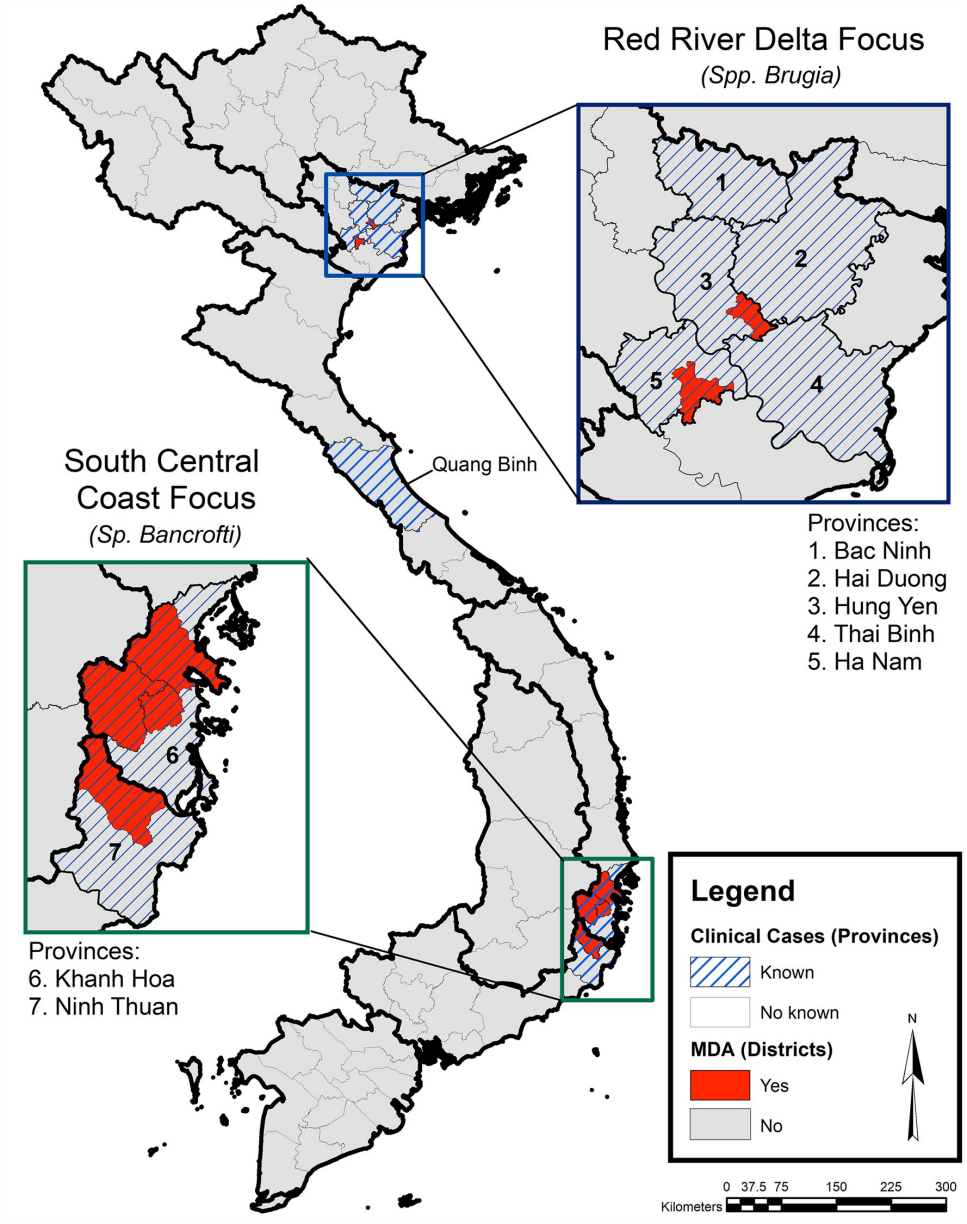
Map of lymphedema patient distribution (province level) and MDA implementation (district level) in Vietnam. Patient information is summarized at the province level in the RRD focus using CHS data collected during 2012 morbidity surveys and data collected during the 2005 MDA in the South Central Coast focus. Information was solicited from Quang Binh province (North Central region) prior to the 2016 DIP survey. The areas in red denote the six districts that conducted MDA in the GPELF era.

At the time of the DIP implementation, patient estimates from five provinces in the RRD region, one province in the North Central region, and two provinces in the South Central Coast were available to NIMPE (Table 2). Patients were reported from 213 CHS from the five provinces in the RRD region, 2 CHS from the province of Quang Binh in North Central region, and 4 CHS from the two provinces in South Central Coast region (Table 2). Among the total 508 patients identified, 26 patients (12%) resided in the six districts that were targeted for MDA during the GPELF era of the LF program. Fig 1 displays the locations of GPELF-era MDA activities as well as the location of reported lymphedema patients.

**Table 2:** Distribution of lymphedema patients by province*.

Region/Province	Districts with lymphedema patients	CHS with lymphedema patients	Number of lymphedema patients
*Red River Delta*			
Bac Ninh	5	19	24
Ha Nam	4	36	173
Hai Duong	12	87	184
Hung Yen	10	30	37
Thai Binh	8	41	61
*North Central*			
Quang Binh	2	2	25
*South Central Coast*			
Khanh Hoa	2	2	2
Ninh Thuan	1	2	2
**Total**	**44**	**219**	**508**

*Patient data for the Red River Delta Region and the South Central Coast Region were from patient estimation surveys in 2012 and 2005 respectively; patient data for the North Central Region were collected during a survey just prior to the 2016 DIP survey.

A total of 32 CHS were surveyed, with number of lymphedema patients served ranging from one to 27 with a median of one. Survey implementation took an average of two hours per CHS; this included an introductory meeting with the CHS director, a health staff interview, a physical inspection of the CHS, and, in some cases, a patient interview. Approximately two CHS were completed per day, including meetings with province and district offices to introduce the survey. The implementation budget was approximately USD$9,600, or USD$300 per facility, which included travel and per diem for two NIMPE, two provincial, two district and CHS staff, as well as hiring two vehicles. This cost did not account for in-kind contributions of staff time and vehicles from the Ministry of Health. It also did not include costs associated with time, travel and per diem for partners such as CDC, RTI International, Helen Keller International and WHO as they were present only as part of the evaluation process and would not be expected to take part in the DIP implementation by other national programs.

### Scores

The average facility score was 8.8 of 14 possible points (63%) and ranged from 4 (29%) to 13 (93%) (S2 Table). The region and national average scores for each of the tracer indicators are listed in Table 3.

**Table 3:** Average indicator scores by region and national, direct inspection protocol, Vietnam, 2016.

Indicators	Red River Delta (n = 24)	North Central (n = 2)	South Central Coast (n = 6)	National Average
1 – Trained staff	0%	0%	0%	**0%**
2 – Lymphedema management guidelines	75%	0%	0%	**56%**
3 – IEC* or awareness materials	21%	0%	0%	**16%**
4 – Improved water infrastructure	92%	100%	100%	**94%**
5 – Antiseptics available	79%	50%	100%	**81%**
6 – Antifungals available	38%	100%	50%	**44%**
7 – Antibiotics available	100%	100%	83%	**97%**
8 – Analgesics available	100%	100%	67%	**94%**
9 – Lymphedema management supplies	100%	100%	100%	**100%**
10 – Patient tracking system	13%	0%	0%	**9%**
11 – Signs/symptoms of lymphedema	75%	100%	0%	**63%**
12 – Lymphedema management strategies	83%	50%	33%	**72%**
13 – Signs/symptoms of lymphedema	83%	100%	67%	**81%**
14 – Acute attack management strategies	75%	50%	83%	**75%**
**Facility Percentage**	**67%**	**61%**	**35%**	

*IEC = information, education and communication materials for lymphedema management

**Fig 2:**
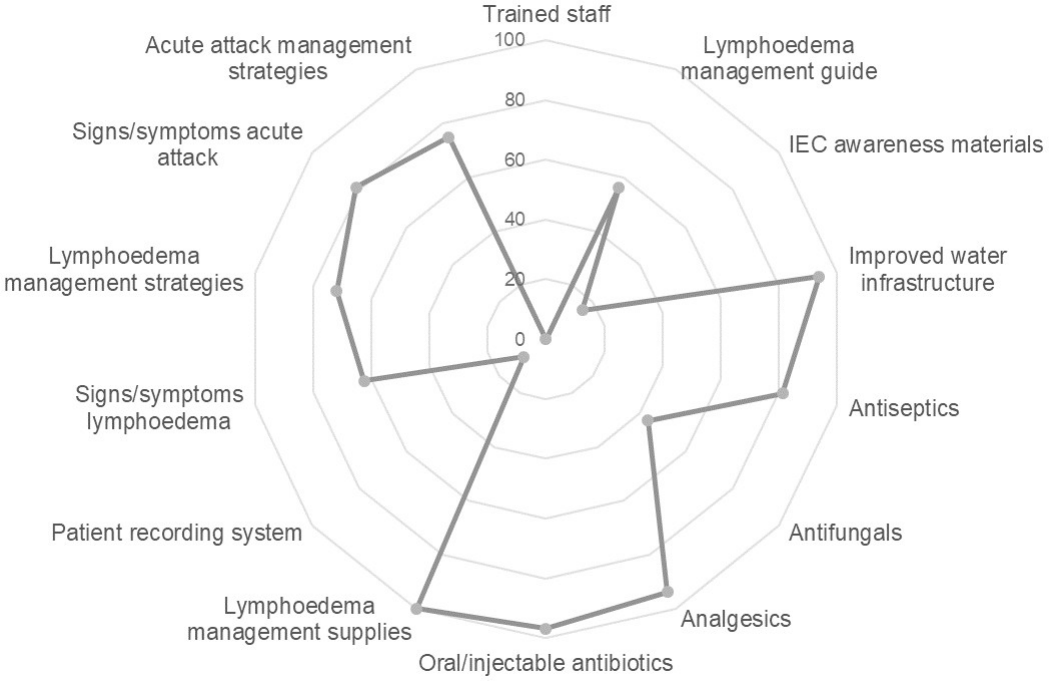
Percentage of facilities (n = 32) achieving quality indicator criteria. Percentage of health facilities nationally meeting the criteria for each of the 14 lymphedema management quality indicators.

The highest scoring indicator, lymphedema management supplies, showed that basic materials to care for lymphedema were available in all facilities (Fig 2). Improved water infrastructure was present in almost all facilities (94%). The medications and commodities needed to provide adequate lymphedema management services, with the exception of antifungal medications, were generally available across the surveyed sites. Scores were lower for the indicators related to recent staff training and patient tracking. Generally, facilities lacked case management guidelines and IEC materials. No staff were trained in the two years prior to the survey, while 8 staff members had ever received training (25%); however, most staff had some basic knowledge of lymphedema diagnosis and management. Regular follow-up of lymphedema patients was not conducted at most health facilities, as evidenced by limited patients documented in a patient tracking system.

### Semi-structured interviews

Twenty- four patients were seen at 22 of the facilities. Of these, 14 patients consented to interviews and data was collected using the questions in the patient interview section of the protocol. The patients interviewed had an average age of 80.1 years (range 62 to 99 years) and 11 (78.6%) were female. All patients reported ever experiencing an acute attack, with 11 (78.6%) reporting no acute attacks in the last 30 days, 2 (14.3%) experiencing a single acute attack, and 1 (7.1%) patient reporting 4 episodes. Most of the patients (n = 10, 71.4%) had been trained in lymphedema management. Eleven (78.6%) of the patients were performing recommended hygiene measures, with 9 of the respondents reporting that they washed their affected limb at least once daily. Twelve patients knew at least one way to manage their lymphedema, most frequently citing hygiene (n = 9; 75.0%) and elevation (n = 4; 33.3%); and eleven knew at least one way to treat acute attacks, although three of these patients suggested treating with traditional herbs only. Nine rated their feelings about lymphedema in past 30 days as ‘good’, ‘moderately good’ or ‘very good’. Of the five who rated their feelings as ‘bad’ or ‘very bad’, two also reported acute attacks in the last month. The majority (n = 12; 85.7%) were satisfied with the lymphedema management services provided at the health facility. The most common suggestion to improve services was for the health facilities to provide patients supplies for lymphedema management (7; 50%), specifically medicines. Other suggestions also included expanding human resources at health facilities (n = 2) and improving training for health facility personnel (n = 2).

Semi-structured interviews were available from staff at 24 of the CHS pertaining to the challenges providing care and suggestions for improving services. Many facilities reported no specific challenges providing services (n = 16), often due to low case counts (n = 5). Those reporting challenges cited limited health seeking behavior (n = 2) or patients bypassing CHS to present at district health facilities (n = 2).

Further, lack of information (n = 2), lack of equipment and medicines (n = 2), poor infrastructure (n = 2), and challenges working with older patients (n = 2) were cited. Even those reporting few challenges for services provision noted that patient economic status (n = 5), specifically lack of health insurance, posed a challenge for patient access. Among suggestions for improvement, training for staff was most commonly cited (n = 12). Other suggestions included improving the availability of medications and commodities (n = 4), lymphedema management materials for patients (n = 2), and IEC materials (n = 2). Further, improving patients’ economic status or providing direct funding to patients (n = 4) was suggested.

## Discussion

NIMPE and the survey teams found the DIP to be a useful and feasible tool to identify facilities and indicators that needed bolstering in order to improve the availability of quality lymphedema management services in Vietnam. At $9,600, the cost of implementing the DIP nationwide was similar to the median cost of a LF transmission assessment survey in an evaluation unit to determine whether MDA can be stopped [13]; the three weeks necessary to implement the DIP was equivalent to the time needed to implement between one to three transmission assessment surveys, depending on districts’ accessibility. Given that each evaluation unit needs to implement three transmission assessment surveys over the course of four-six years to obtain data for the elimination dossier, this seems to be a reasonable allocation of time and resources for collection of MMDP availability data. Along with providing useful and actionable information, the questionnaire was considered by NIMPE staff to be easy to administer and the use of electronic data collection tools ensured high quality data and facilitated completion of analyses in a timely manner.

At the national scale, the CHS surveyed were found to be well equipped to treat lymphedema patients, and generally had the necessary infrastructure and commodities to provide lymphedema management services. Not surprisingly, since the LF program had not implemented formal MMDP activities after 2012, trained health staff and depletion of case management and education materials were common areas of weakness across facilities. Staff turnover was identified by health facilities as a particular challenge, whereby staff from many health facilities had been trained previously but had either retired or no longer worked at the facility.

The low rate of patient tracking was likely due to tracking and follow up of lymphedema patients not being an explicit priority, either in the health information system or in the LF program reporting requirements. This was in contrast to the leprosy program which issued a government circular asking districts to report on a set of case finding and patient tracking leprosy elimination indicators recommended by WHO [14, 15]. Anecdotal information from the semi-structured interviews also noted that the health seeking behavior of patients with lymphedema was low; these persons may present to the CHS for other health issues, but not for lymphedema, mostly because they hadn’t experienced acute attacks in recent years.

In the RRD region, the facility quality scores were similar across CHS and higher than the scores seen in the other regions in Vietnam. This is likely because these provinces were previously targeted for MMDP activities due to the high burden of persons with lymphedema (Table 2).

In response to these findings, NIMPE took several actions to improve the quality of lymphedema management services nationally. First, in response to the lack of recent training, the LF national program conducted training courses in RRD region in 2016 and 2017 for over 397 health staff at provincial, district and CHS levels in 14 provinces to strengthen lymphedema care capacity. Further, to address the lack of case management and education materials, NIMPE produced and disseminated updated health facility posters and patient flyers. Finally, to address the lack of patient tracking, NIMPE distributed patient tracking registers to CHS staff, based on leprosy patient registers that were shared with the survey team during the direct inspection visits. The leprosy patient registers were used as a prototype since they were familiar to commune health staff. Commune health staff were asked to register all patients and follow them regularly. In Quang Binh (Central), and Khanh Hoa and Ninh Thuan (South Central coast) provinces, due to the small volume of patients LF program national staff trained CHS staff and patients directly and disseminated hygiene and educations materials.

This initial experience with the DIP provides important lessons for the implementation of readiness assessments for lymphedema management in other settings. First, national programs should ensure that all areas that historically reported patients with clinical manifestations of LF are included in the patient estimation and data collection exercises, as highlighted by our findings that 88% of identified patients were living outside areas that required MDA. Furthermore, the sooner national programs can compile data on estimated number of patients per IU, the more easily they can plan and work within the health system to ensure implementation of the recommended services. It was noted that on-site visits to health facilities and direct communication with health facility staff and community members provided an opportunity to identify patients previously unknown to the national or provincial/district program.

Second, integrating the protocol implementation with data collection efforts for other health programs with geographic and thematic overlaps, such as leprosy, non-communicable diseases, and rehabilitation services, is a possible adaptation that could improve the feasibility of survey implementation. As has been done with community case finding activities for skin diseases [16], integrating questions on similar themes of patient hygiene and follow-up into the direct inspection protocol for leprosy or diabetes programs could reduce some fragmentation of health facility data collection but still provide specific information. In addition to cost-savings, this integration would offer an opportunity to look at quality of lymphedema services in the context of universal health coverage, recognizing that these services need to be maintained after elimination of LF. In Vietnam, integration between the national leprosy and LF programs presents an opportunity for collaboration that may ensure sustainability of care for persons with lymphedema.

Finally, while this survey was used to evaluate the quality of lymphedema management services just prior to requesting validation of the elimination of LF as a public health problem in Vietnam, the protocol could be used to assess the baseline level of quality of lymphedema management services to refine implementation efforts. Further, countries should be encouraged to use this tool as an interim assessment to track the expansion of MMDP services and ensure the availability of quality lymphedema management services for all persons with lymphedema.

In addition to assessing the readiness of health facilities to provide MMDP services, lymphedema patient estimation efforts may identify areas with residual infection that would otherwise go unnoticed. Since lymphedema patients may reside outside of IUs implementing MDA, assessment of LF MMDP services may be conducted in areas classified as non-endemic that are not targeted for other LF-related surveys. The identification of lymphedema patients conducted as part of this exercise led to the recognition of a possible location of ongoing infection in the province of Quang Binh. Historically, Quang Binh exhibited among the highest burden of LF infection in Vietnam, second only to the province of Ha-Nam [11]. However, this area did not require MDA based on the 2003 mapping data. Given the number of lymphedema patients identified as part of this survey, including one patient as young as 16 years of age, NIMPE implemented confirmatory mapping of those areas in April 2017, following a protocol developed by the NTD Support Center [17]. Because no positive LF antigen or antibody results were detected among children in grades 4 and 5 in those districts, MDA was not recommended.

While there are a variety of methods available to assess readiness of facilities to provide quality care, including the WHO Systems Availability and Readiness Assessment, the Service Provision Assessment, and the World Bank’s Service Delivery Indicators tools [8, 18, 19], these and other health facility assessment tools contain different indicators and use varying methodologies, which has led to gaps in comparability and in linking availability of services to outcomes [20–22]. In addition, they do not include NTD-specific indicators. This protocol provides a tested methodology including specific lymphedema management questions that can be easily adapted by national LF programs. As a result of this evaluation, the protocol was updated by making minor revisions to language and finalizing the scoring system and the follow-up recommendations and will be released by WHO as part of their LF MMDP Toolkit.

National programs can further adapt it to country contexts, for example, by adding an indicator to assess sustainability of self-care kits in areas where kits were given out to patients. In addition, programs could collect data on payments for services, given the new WHO 2030 roadmap goal of 100% of endemic countries are providing LF interventions without out-of-pocket expense for the patients, with the caveat that studies have found these data difficult to collect [8].

A challenge of this study was the varying reliability and quality of patient data, as patient estimates were based on self-reporting from provinces and did not follow a standardized approach. It is likely that patients were missed in this exercise. The availability of robust patient burden information is critical to ensure that the sampling framework of health facilities that should be providing services is complete; however, this is likely challenging in many settings due to the difficulty of identifying and tracking persons with lymphedema.

Further research is needed to determine whether data saturation can be achieved with a smaller sample size and to assess how the questionnaire could be combined with other NTD or health activities, such as LF transmission assessment surveys or other health facility supervision visits. In addition, research to more clearly understand the relationships between service availability and access to lymphedema management services, the practice of ongoing self-care activities, and patient outcomes could help clarify if other indicators should be added to the protocol.

Overall, the DIP is a simple, flexible, and user-friendly tool to help countries rapidly identify and respond to weaknesses in providing quality lymphedema management services in all areas with persons with lymphedema. The experience in Vietnam demonstrated that results of this survey can yield actionable suggestions to strengthen MMDP services in order to comply with WHO’s recommended minimum package of care. In 2018, the results of this survey helped to support the claim that Vietnam had eliminated LF as a public health problem, which was subsequently validated by the World Health Organization.

## Supporting information

S1 Table Suggested action items. List of possible action items following the direct inspection protocol.
(DOCX)

S2 Table Vietnam direct inspection protocol survey results. Facility-level direct inspection protocol results for Vietnam.
(DOCX)

S1 STROBE Checklist STROBE checklist. (DOC)
