# Supplementary material for: Evaluation of a facility-based inspection tool to assess lymphedema management services in Vietnam
Source: PLoS Negl Trop Dis. 2020 Oct 19;14(10):e0008773. doi: 10.1371/journal.pntd.0008773 (PMC7595627; doi:10.1371/journal.pntd.0008773)
Supplement: S1 Table — List of possible action items following the direct inspection protocol. (DOCX) [file pntd.0008773.s001.docx]

**Supplemental Table 1. Suggested Action Items by Indicator Based on Results of Direct Inspection Protocol**

| Indicators | Actions |
| --- | --- |
| Trained Staff (Indicator 1) | - Use quantitative and qualitative methods to help identify the specific challenges with training (e.g. staff turnover, failed cascade training) - Organize additional training sessions, especially if no trainings have been given in last 2 years - Consider expanding number of individuals trained per facility and review procedures for staff turnover - Incorporate MMDP messages into medical and nursing curriculum - Refer to the [WHO MMDP toolkit](http://www.who.int/lymphatic_filariasis/global_progress/managing_morbidity_preventing_disability_toolkit/en/) for tools to assist with staff training |
| Case Management and Education Materials (Indicators 2-3) | - Work with local health education partners and local artists to adapt existing materials or create new materials - Secure funding for duplication of case management and education materials - Ensure materials are available in sufficient quantities at facilities providing MMDP services - Refer to the [WHO MMDP toolkit](http://www.who.int/lymphatic_filariasis/global_progress/managing_morbidity_preventing_disability_toolkit/en/) for examples of case management and education materials |
| Water Infrastructure (Indicator 4) | - Identify specific issues related to water access (e.g. type of infrastructure, service interruptions, distance of infrastructure from facility) - Disseminate results of survey to relevant organizations (e.g. Ministry of Water Resources, Ministry of Health) - Advocate for improved health care facility infrastructure, including provision of water infrastructure - Influence and/or develop partnerships with existing WASH initiatives (e.g. water safety plans, protocol for water and health, etc.) - Encourage WASH and NTD joint strategic planning - Refer to [Water, sanitation, and hygiene in health care facilities: Status in low- and middle-income countries and way forward for more information on WASH in health care facilities](http://apps.who.int/iris/bitstream/10665/154588/1/9789241508476_eng.pdf?ua=1) for more information on WASH in health care facilities - Refer to [Water sanitation and hygiene for accelerating and sustaining progress on neglected tropical diseases: A global strategy 2015-2020](http://www.who.int/water_sanitation_health/publications/wash-and-ntd-strategy/en/) for more information on mobilizing WASH and NTD actors to work together towards roadmap targets |
| Medications and Commodities (Indicators 5-9) | - Improve policies and procedures for supply chain management and address identified gaps - Identify supplies outside of normal services that require additional investment - Secure a regular shipment of supplies - Identify and access fund sources needed to maintain access to needed supplies - Liaise with institutions responsible for supply management at health care facilities |
| Patient Tracking System (Indicator 10) | - Develop a system for tracking patients, either in paper or electronic format, as appropriate for the setting |
| Staff Knowledge (Indicators 11-14) | - Determine if additional training is required (see Trained Staff). - Review training materials to evaluate appropriateness and comprehension of materials (e.g. consider language, etc.) - Evaluate the success of previous training sessions and diffusion of key messages - Work to incorporate MMDP messages into standard medical and nursing curricula |
