# Supplementary material for: Evaluation of a facility-based inspection tool to assess lymphedema management services in Vietnam
Source: PLoS Negl Trop Dis. 2020 Oct 19;14(10):e0008773. doi: 10.1371/journal.pntd.0008773 (PMC7595627; doi:10.1371/journal.pntd.0008773)
Supplement: S2 Table — Facility-level direct inspection protocol results for Vietnam. (DOCX) [file pntd.0008773.s002.docx]

**Supplemental Table 2. Vietnam direct inspection protocol survey results.**

| **Facility** | **Indicators** | | | | | | | | | | | | | | **Facility Score** | **Facility Percentage** |
| --- | --- | --- | --- | --- | --- | --- | --- | --- | --- | --- | --- | --- | --- | --- | --- | --- |
|  | **1** | **2** | **3** | **4** | **5** | **6** | **7** | **8** | **9** | **10** | **11** | **12** | **13** | **14** |  |  |
| **Red River Delta Region** |  |  |  |  |  |  |  |  |  |  |  |  |  |  |  |  |
| **1** | 0 | 1 | 1 | 0 | 0 | 0 | 1 | 1 | 1 | 0 | 1 | 1 | 1 | 1 | 9 | 64.3% |
| **2** | 0 | 1 | 1 | 1 | 1 | 0 | 1 | 1 | 1 | 0 | 1 | 1 | 1 | 1 | 11 | 78.6% |
| **3** | 0 | 0 | 0 | 1 | 1 | 0 | 1 | 1 | 1 | 1 | 1 | 0 | 0 | 1 | 8 | 57.1% |
| **4** | 0 | 1 | 0 | 1 | 1 | 0 | 1 | 1 | 1 | 0 | 0 | 1 | 0 | 0 | 7 | 50.0% |
| **5** | 0 | 1 | 0 | 1 | 1 | 1 | 1 | 1 | 1 | 0 | 1 | 1 | 1 | 1 | 11 | 78.6% |
| **6** | 0 | 1 | 0 | 1 | 1 | 0 | 1 | 1 | 1 | 0 | 0 | 1 | 1 | 1 | 9 | 64.3% |
| **7** | 0 | 1 | 0 | 1 | 1 | 0 | 1 | 1 | 1 | 0 | 1 | 1 | 1 | 0 | 9 | 64.3% |
| **8** | 0 | 1 | 0 | 1 | 1 | 1 | 1 | 1 | 1 | 0 | 1 | 1 | 1 | 1 | 11 | 78.6% |
| **9** | 0 | 0 | 0 | 1 | 1 | 0 | 1 | 1 | 1 | 0 | 1 | 1 | 1 | 1 | 9 | 64.3% |
| **10** | 0 | 1 | 0 | 1 | 1 | 1 | 1 | 1 | 1 | 0 | 1 | 1 | 1 | 0 | 10 | 71.4% |
| **11** | 0 | 1 | 0 | 1 | 1 | 0 | 1 | 1 | 1 | 0 | 1 | 1 | 1 | 1 | 10 | 71.4% |
| **12** | 0 | 0 | 0 | 1 | 1 | 1 | 1 | 1 | 1 | 0 | 1 | 1 | 1 | 1 | 10 | 71.4% |
| **13** | 0 | 1 | 0 | 1 | 1 | 0 | 1 | 1 | 1 | 0 | 1 | 1 | 1 | 1 | 10 | 71.4% |
| **14** | 0 | 0 | 0 | 1 | 1 | 1 | 1 | 1 | 1 | 0 | 1 | 0 | 1 | 0 | 8 | 57.1% |
| **15** | 0 | 0 | 0 | 1 | 1 | 1 | 1 | 1 | 1 | 0 | 1 | 1 | 1 | 1 | 10 | 71.4% |
| **16** | 0 | 1 | 1 | 1 | 1 | 1 | 1 | 1 | 1 | 1 | 1 | 1 | 1 | 1 | 13 | 92.9% |
| **17** | 0 | 1 | 1 | 1 | 0 | 0 | 1 | 1 | 1 | 0 | 0 | 1 | 1 | 1 | 9 | 64.3% |
| **18** | 0 | 1 | 1 | 1 | 1 | 1 | 1 | 1 | 1 | 0 | 1 | 1 | 1 | 1 | 12 | 85.7% |
| **19** | 0 | 1 | 0 | 0 | 1 | 0 | 1 | 1 | 1 | 0 | 1 | 1 | 1 | 1 | 9 | 64.3% |
| **20** | 0 | 1 | 0 | 1 | 0 | 0 | 1 | 1 | 1 | 1 | 1 | 1 | 1 | 1 | 10 | 71.4% |
| **21** | 0 | 1 | 0 | 1 | 0 | 0 | 1 | 1 | 1 | 0 | 0 | 1 | 1 | 1 | 8 | 57.1% |
| **22** | 0 | 1 | 0 | 1 | 0 | 0 | 1 | 1 | 1 | 0 | 0 | 0 | 0 | 0 | 5 | 35.7% |
| **23** | 0 | 0 | 0 | 1 | 1 | 1 | 1 | 1 | 1 | 0 | 0 | 0 | 1 | 0 | 7 | 50.0% |
| **24** | 0 | 1 | 0 | 1 | 1 | 0 | 1 | 1 | 1 | 0 | 1 | 1 | 0 | 1 | 9 | 64.3% |
| **South Central Coast** |  |  |  |  |  |  |  |  |  |  |  |  |  |  |  |  |
| **25** | 0 | 0 | 0 | 1 | 1 | 1 | 1 | 0 | 1 | 0 | 0 | 0 | 1 | 0 | 6 | 42.9% |
| **26** | 0 | 0 | 0 | 1 | 1 | 0 | 0 | 0 | 1 | 0 | 0 | 0 | 0 | 1 | 4 | 28.6% |
| **27** | 0 | 0 | 0 | 1 | 1 | 1 | 1 | 1 | 1 | 0 | 0 | 1 | 1 | 1 | 9 | 64.3% |
| **28** | 0 | 0 | 0 | 1 | 1 | 1 | 1 | 1 | 1 | 0 | 0 | 0 | 1 | 1 | 8 | 57.1% |
| **29** | 0 | 0 | 0 | 1 | 1 | 0 | 1 | 1 | 1 | 0 | 0 | 1 | 1 | 1 | 8 | 57.1% |
| **30** | 0 | 0 | 0 | 1 | 1 | 0 | 1 | 1 | 1 | 0 | 0 | 0 | 0 | 1 | 6 | 42.9% |
| **North Central** |  |  |  |  |  |  |  |  |  |  |  |  |  |  |  |  |
| **31** | 0 | 0 | 0 | 1 | 1 | 1 | 1 | 1 | 1 | 0 | 1 | 0 | 1 | 0 | 8 | 57.1% |
| **32** | 0 | 0 | 0 | 1 | 0 | 1 | 1 | 1 | 1 | 0 | 1 | 1 | 1 | 1 | 9 | 64.3% |
|  |  |  |  |  |  |  |  |  |  |  |  |  |  |  |  |  |
| **National Average** | 0% | 56% | 16% | 94% | 81% | 44% | 97% | 94% | 100% | 9% | 63% | 72% | 81% | 75% |  |  |
